# Effect of interfractional shoulder motion on low neck nodal targets for patients treated using volumetric‐modulated arc therapy (VMAT)

**DOI:** 10.1120/jacmp.v16i4.5206

**Published:** 2015-07-08

**Authors:** Kevin E. Casey, Pei‐Fong Wong, Samuel S. Tung

**Affiliations:** ^1^ Department of Radiation Physics University of Texas MD Anderson Cancer Center Houston TX USA

**Keywords:** VMAT, shoulders, motion, supraclavicular, dose

## Abstract

VMAT is an important tool in the treatment of head and neck cancers, many of which also require treatment to the supraclavicular lymph nodes. However, full VMAT arcs treating this nodal region necessarily cause entrance beam to pass through patients' shoulders. Thus, interfractional variations in shoulder position may cause unwanted dose perturbations. To assess this possibility, six patients undergoing treatment at our institution for head and neck cancers with associated supraclavicular lymph node treatment were imaged with in‐room CT‐on‐rails during the course of their treatments. This allowed for the establishment of a true record of the actual shoulder position during selected treatment fractions. Then, a full VMAT plan and a plan with VMAT arcs superior to the shoulder and a static anteroposterior field inferiorly were copied onto the patients' weekly image sets. The average one‐dimensional shoulder motion was generally within 10 mm of the simulated position, with some notable exceptions. The standard deviation in week‐to‐week shoulder position relative to simulation was 4.3 mm and 4.2 mm in the SI and AP dimensions, respectively. The average nodal target mean dose across all fractions sampled was within 5% of planned for all patients and both plans. Similarly, the average D95 for the nodal target was within 5% of planned across all fractions sampled, with the single exception of the full VMAT plan for one patient. In most cases, the standard deviation in both target mean dose and D95 was smaller with the VMAT+static AP field plan than it was with the full VMAT plan.

PACS number: 87.55.‐x

## I. INTRODUCTION

Volumetric‐modulated arc therapy (VMAT)^(1)^ has become an important technique in radiation oncology, including in the treatment of head and neck cancers. The use of VMAT in head and neck treatments may offer a combination of reduced treatment time, better organ‐at‐risk (OAR) sparing, or increased target dose conformality compared to other treatment types.^2^, ^3^, ^4^, ^5^ Many head and neck treatment plans include regional lymph nodes in the supraclavicular area. A conventional 360° VMAT arc intended to treat these nodal targets will necessarily cause the entrance beam to pass directly through the patient's shoulders. Therefore, interfractional shoulder position changes could potentially cause clinically significant dose perturbations when using conventional arcs.

Neubauer et al.^(6)^ investigated the effect of interfractional shoulder motion on supraclavicular targets using planning structures and found potential for loss of target coverage or increased OAR dose when treating with full 360° VMAT arcs. However, in‐room CT‐on‐rails technology in our institution allows us to obtain weekly or daily diagnostic‐quality CT scans of patients in the actual treatment position immediately prior to treatment delivery. The CT‐on‐rails system consists of a diagnostic‐quality fan beam CT scanner (GE Healthcare, Waukesha, WI) in the same room as our Varian 21EX linear accelerator (Varian Medical Systems, Palo Alto, CA). The two units share a common patient couch, which may be rotated through 180°. In the rotated couch position, the CT scanner can slide along rails installed in the floor in order to translate the patient through the bore and obtain an image. Thus, a diagnostic‐quality CT scan can be acquired after the patient has been set up for treatment. By obtaining CT scans using this system periodically throughout the course of treatment, we may obtain a true record of the patient's interfractional shoulder motion and use our treatment planning system (Pinnacle 9.4, Philips, Fitchburg, WI) to investigate the actual effect as VMAT arcs pass through the shoulder.

## II. MATERIALS AND METHODS

Six head and neck patients imaged with CT‐on‐rails during their treatment were selected for this study. Three of the patients received treatment to the nasopharynx, two to the base of tongue, and one to the oropharynx. In addition to treatment at the primary site, all six patients required treatment to nodal regions in the supraclavicular area. Furthermore, the treating physicians ordered in‐room CT‐on‐rails alignment verification at least once per week during each patient's treatment.

Each patient was originally planned with a step‐and‐shoot IMRT technique using Pinnacle. These treatment plans consisted of nine IMRT beams spaced at gantry angles 40° apart. These IMRT fields treated the primary CTV and were matched inferiorly to a static anterior–posterior (AP) supraclavicular field intended to treat the nodal target. Each supraclavicular field included a laryngeal block and was followed sequentially by a boost field which was identical, except that the midline was completely blocked. Finally, these fields were followed sequentially by a cone‐down boost to the highest risk area.

It is common in our institution for physicians to delineate a block for the supraclavicular fields rather than contouring actual nodal targets. For this reason, we used the clinical fixed‐beam IMRT treatment plans described above as a guide for contouring supraclavicular nodal targets for each patient. The target contours followed the isodose lines of the original prescription doses. Then, two new treatment plans were created for each patient using Pinnacle. The first plan was a “Full VMAT” plan, in which a pair of VMAT arcs each completely encompassed both the primary and supraclavicular target volumes. Each arc consisted of a full 360° gantry rotation. One arc moved the gantry clockwise and one counterclockwise. In one arc, the collimator was rotated 10° and 350° in the other. The plan was optimized primarily for target coverage uniformity with some additional normal tissue constraints, but no special consideration was given to avoidance of the patients' shoulders. A planning structure expansion of 7 mm in all directions, except superiorly, was used to achieve uniform dose to the nodal target in the case of the Full VMAT plan. In the superior direction, the nodal target contours abutted the physician‐delineated low‐risk neck targets, so no planning structure was necessary in this direction.

**Figure 1:**
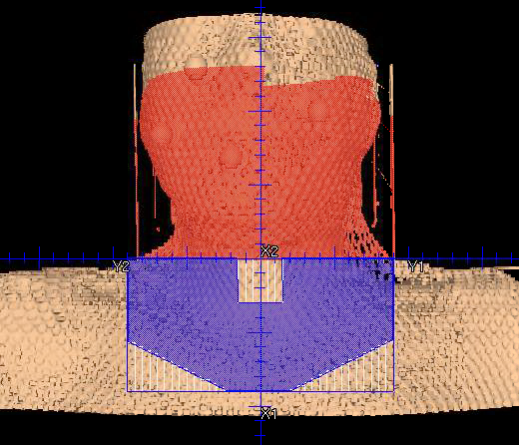
A typical AP supraclavicular field (blue) as found in the Half‐Beam VMAT plan. The field is blocked inferior to the clavicle and over the larynx. The VMAT field is shown in red colorwash. There is no overlap at the match line.

The second plan generated for each patient was a “Half‐Beam VMAT” plan (Fig. 1). These plans were created by combining two 360° VMAT arcs with a static anterior–posterior supraclavicular field based on that of the original IMRT plan. The stop and start angles of the two arcs were separated by 2° with control points every 4°. For the VMAT arcs, the collimator was rotated 90° and the inferior jaw closed to zero. Similarly, the collimator was rotated 90° and the superior jaw was closed for the AP supraclavicular field. In this way the edges of the VMAT arcs and the supraclavicular field were matched with no overlap. The junction point between the fields was set at isocenter in the patient's neck, in the vicinity of C5‐C6. Thus, in this plan, the VMAT arcs were entirely superior to the level of the patients' shoulders. It was ensured that the block included at least 7 mm of margin around the nodal target contours. Any midline‐blocked or sequential boost subfields included in the original IMRT plan were used in the Half‐Beam VMAT plan, as well. Thus the Half‐Beam VMAT plan was analogous to the clinical IMRT plan, with VMAT arcs taking the place of fixed IMRT beam angles. Both the Full VMAT and Half‐Beam VMAT plans were optimized with the goal of creating clinically reasonable plans; however, neither was reviewed by a physician.

**Figure 2:**
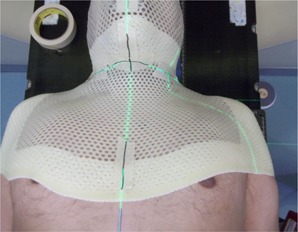
The custom thermoplastic mask used for setup at simulation and treatment. Isocenter is marked in the neck.

The patients were simulated using our normal clinical procedure, which includes a custom five‐point thermoplastic mask (Orfit Industries, Antwerp, Belgium) and head cradle for immobilization (Fig. 2). The mask covered the entire head and extended inferiorly past the shoulders to cover the upper arm and torso to the level of approximately T6. Each patient was treated using the same setup and a Varian 21EX linear accelerator with Millennium 120‐leaf multileaf collimator (Varian).

During the course of treatment, the setup and alignment of each patient was verified with in‐room, CT‐on‐rails at least once per week. The CT images were acquired with a 50 cm field of view and a slice thickness of 2.5 mm. The scan extent was from the top of the orbits to approximately T4. The isocenter was marked on the patient with plastic BBs in order to identify its position on the resulting CT images.

Each patient's weekly CT‐on‐rails images were imported into custom in‐house alignment software.^(7)^ A rigid 3D registration was created between the weekly images and the planning CT using an automatic registration algorithm.^(8)^ A mesh deformation was applied to the original contours so that they best matched the same anatomy in the weekly and simulation images. The deformed contours were evaluated visually to ensure that they did not extend outside the patients' bodies and that they enclosed anatomy comparable to that of the original contours. The alignment software also provides numerical values for four parameters used in optimizing the alignment. In all cases, these values were within ranges that would be considered clinically acceptable at our institution.

The weekly CT images were then imported into our treatment planning system. First, measurements were made of the patients' shoulder position relative to the treatment isocenter, which was identified on the weekly CT images using the BBs affixed during treatment setup. The separation from the humeral head to the treatment isocenter was measured on the CT images separately in the superior–inferior (SI) and anterior–posterior (AP) dimensions. The measured distances were compared to the same distances measured on the original planning CT images in order to quantify the patients' weekly shoulder positioning relative to the simulated position.

Next, the newly‐created Full VMAT and Half‐Beam VMAT plans, plus the deformed contours from our in‐house registration software, were copied onto the weekly CT images. The dose was calculated using a heterogeneous convolution/superposition algorithm. The mean dose to the nodal target was recorded for comparison, as was the dose to 95% of the nodal target volume.

## III. RESULTS

### A. Shoulder motion

**Figure 3:**
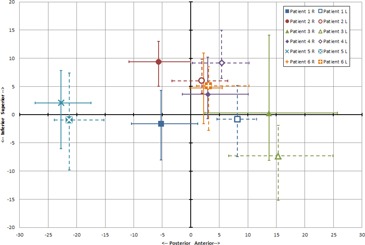
Weekly shoulder positions for the right (R) and left (L) shoulders, relative to the position at time of simulation. Points represent the average position and error bars represent the range.

Weekly shoulder motion relative to simulation is summarized in Table 1 and Fig. 3. In both instances, the given shifts are one‐dimensional and are relative to the position of the shoulders during simulation. Positive numbers indicate shifts in the superior or anterior direction, whereas negative numbers indicate shifts in the inferior or posterior direction.

**Table 1:** Weekly patient shoulder position relative to simulation. Positive numbers indicate a position superior or anterior to the simulated position, negative numbers indicate inferior or posterior. All numbers are in mm

		*Superior(+)–Inferior(‐)*		*Anterior(+)–Posterior(‐)*	
*Patient*	*Patient Shoulder*	*Average*	*SD*	*Average*	*SD*
1	Right	‐1.6	3.7	‐5.2	4.2
	Left	‐0.8	4.0	8.2	2.5
2	Right	9.4	2.9	‐5.6	5.9
	Left	6.0	2.3	1.9	3.2
3	Right	0.3	8.0	13.7	7.9
	Left	‐7.3	5.1	15.3	6.6
4	Right	3.6	3.6	3.0	4.1
	Left	9.2	3.2	5.4	3.9
5	Right	2.1	4.0	‐22.8	4.0
	Left	‐0.9	5.7	‐21.3	2.9
6	Right	4.7	4.6	2.3	1.8
	Left	5.1	3.8	3.1	3.0

The average shoulder position during weekly setup was within 10 mm of the position at simulation in each of the two dimensions considered, with the exception of the anterior–posterior dimension for Patient 3 and Patient 5. Across all patients, the standard deviation of shoulder motion in the superior–inferior and anterior–posterior directions were comparable at 4.3 mm and 4.2 mm, respectively.

In some cases, an unusual shoulder position at simulation introduced a systematic deviation in the weekly position compared to simulation. The most striking example is the consistent posterior shift in Patient 5's shoulders at weekly setup as compared to simulation. Other examples are the shoulders of Patient 2, which were consistently superior to the simulated position in weekly images, and the shoulders of Patient 3 which were consistently anterior to the simulated position.

### B. Nodal target deformations

Size information for each patient's nodal target volume is given in Table 2. The nodal targets ranged in size from $80.3\;{\text{cm}}^{3}$ to $347.9\;{\text{cm}}^{3}$ at simulation. Weekly data are the average values across all fractions sampled for each patient. The images were aligned and the contours were deformed individually for each fraction sampled in the study.

**Table 2:** Nodal target contour size at simulation and after deformation based on weekly images. Weekly figures are given as an average of all fractions sampled for the given patient plus or minus 1 SD

	*Volume* (${\text{cm}}^{3}$)		*Maximum SI Extent (cm)*	
*Patient*	*Simulation*	*Weekly*	*Simulation*	*Weekly*
1	157.9	154.0±5.2	6.25	6.41±0.1
2	283.1	261.9±11.7	7.75	8.17±0.3
3	347.9	313.1±15.8	8.00	8.43±0.2
4	171.0	170.7±12.8	7.50	7.82±0.3
5	80.3	74.2±3.1	4.75	4.19±0.1
6	244.1	207.3±11.2	10.25	8.94±0.4

#### C.1 Mean dose

**Figure 4:**
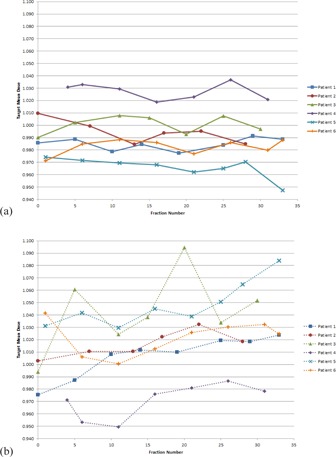
Mean dose to the nodal target at each weekly fraction sampled, using the Half‐Beam (a) and Full VMAT (b) plans. Target mean dose is normalized to the mean dose on the original simulation plan. Markers represent a sampled fraction and lines are merely present to guide the eye. “Fraction 0” signifies a “dry run”, in which the patient was set up and imaged but not actually treated.

**Figure 5:**
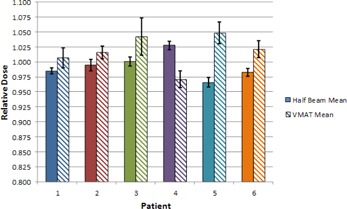
Average nodal target mean dose across all fractions sampled for each patient. The dose is normalized to the original simulation plan. Error bars represent 1 SD.

The target mean dose for the nodal target is shown in Fig. 4 and summarized in Fig. 5. All doses were normalized to the target mean dose calculated on the patients' original simulation CT scans. In Fig. 4, “Fraction 0” signifies a “dry‐run” on the day before normal treatment was to begin, in which the patient was fully set up and imaged, but not actually treated.

In general, the weekly mean dose to the nodal target was within 5% of planned, with a few exceptions. The Full VMAT plan resulted in a target mean dose more than 5% higher than planned for 3 of 7 and 3 of 8 fractions sampled for Patient 3 and Patient 5, respectively. Patient 4 had 1 weekly Full VMAT fraction (out of 7 sampled) in which the target mean dose was 5.1% lower than planned. Patient 5 had 1 weekly Half‐Beam VMAT fraction (out of 8 sampled) in which the target mean dose was 5.2% lower than planned.

Target mean dose information is presented in Table 3. For the six patients, the average normalized target mean dose across all fractions ranged from 0.966 to 1.028 for the Half‐Beam VMAT plan and 0.971 to 1.048 for the Full VMAT plan. Notably, with the exception of Patient 2, the standard deviation in the target mean dose using the Full VMAT plan was at least double that of the Half‐Beam VMAT plan. For Patient 2, the Full VMAT plan standard deviation was only slightly higher than that of the Half‐Beam VMAT plan.

**Table 3:** Average nodal target mean dose across all fractions sampled for each patient

	*Half‐Beam VMAT*		*Full VMAT*	
*Patient*	*Average*	*SD*	*Average*	*SD*
1	0.985	0.005	1.007	0.017
2	0.995	0.009	1.016	0.010
3	1.001	0.007	1.043	0.031
4	1.028	0.007	0.971	0.014
5	0.966	0.008	1.048	0.018
6	0.983	0.006	1.022	0.014
Average	0.993	0.007	1.018	0.018

#### C.2 D95

**Figure 6:**
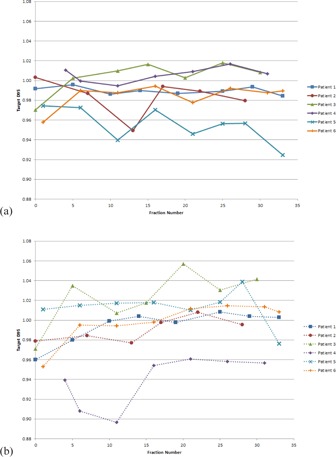
Dose to 95% of the nodal target at each weekly fraction sampled, using the Half‐Beam (a) and Full VMAT (b) plans. Weekly D95 is normalized to D95 on the original simulation plan. Markers represent a sampled fraction and lines are merely present to guide the eye. “Fraction 0” signifies a “dry run”, in which the patient was set up and imaged but not actually treated.

**Figure 7:**
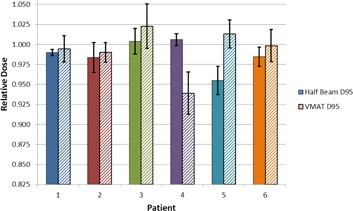
Average nodal target D95 across all fractions sampled for each patient. The dose is normalized to the original simulation plan. Error bars represent 1 SD.

The dose that covers 95% of the nodal target volume, known as D95, for each fraction sampled is shown in Fig. 6 and summarized in Fig. 7. For most sampled fractions, D95 was within 5% of planned. With the Full VMAT plan, Patient 3 had 1 of 7 fractions more than 5% higher than planned, and Patient 4 had 3 of 7 fractions more than 5% lower than planned.

With the Half‐Beam VMAT plan, Patient 2 had 1 fraction out of 6 sampled at 5% lower than planned, and Patient 5 had 3 out of 8 more than 5% lower than planned. For all other fractions sampled in the study, D95 was within 5% of planned for the Half‐Beam VMAT plan.

Nodal target D95 data are presented in Table 4. As with mean dose, the standard deviation in D95 among weekly fractions sampled was generally lower for the Half‐Beam VMAT plan than for the Full VMAT plan. The exception was Patient 2. For Patient 5, the two values were comparable, with a Half‐Beam VMAT standard deviation of 0.018 compared to a Full VMAT standard deviation of 0.017.

**Table 4:** Average nodal target D95 across all fractions sampled for each patient

	*Half‐Beam VMAT*		*Full VMAT*	
*Patient*	*Average*	*SD*	*Average*	*SD*
1	0.990	0.004	0.994	0.016
2	0.984	0.019	0.990	0.012
3	1.004	0.016	1.023	0.028
4	1.006	0.007	0.939	0.026
5	0.955	0.018	1.013	0.017
6	0.985	0.012	0.999	0.020
Average	0.987	0.013	0.993	0.020

### D. Treated volume

The volume of normal tissue in the low neck (inferior to the isocenter) receiving at least 45 Gy (V45) in each plan was measured on the original simulation scans. The V45 for the Half‐Beam plan averaged 75.2% of that of the Full VMAT plan for all six patients (range 53.4%–99.9%). It is important to note that the Full VMAT plans were optimized primarily for target coverage, with lesser consideration given to normal tissue sparing.

## IV. DISCUSSION

Neubauer et al.^(6)^ did not observe any changes in shoulder position greater than 20 mm in any dimension in a sample of 10 patients using the same immobilization device. However, we did observe some anterior shifts for Patient 3 and posterior shifts for Patient 5 exceeding 20 mm. This may indicate that these patients were simulated in a less reproducible position. Aside from these outliers, the shifts we observed were more consistent with the 2–6 mm average found by Neubauer and colleagues. The precision of our shoulder measurements was degraded by the finite CT scan slice thickness (2.5 mm) and the time delay between CT imaging and treatment, during which the table was physically rotated through 180°. The limited size of this study (six patients) restricts our ability to draw conclusions about the interfractional shoulder motion of the overall patient population. It is possible that the small sample we investigated either over‐ or underestimates the median and average shoulder motion with our immobilization device.

The overall change in mean dose from planned to that calculated on weekly CT images is given in Table 5. This number was calculated by multiplying the weekly average target mean dose per fraction (Table 3) by a hypothetical 60 Gy prescription dose. All changes were within 3 Gy.

It is notable that the Full VMAT plan resulted in a higher average relative dose than the Half‐Beam plan in all cases, with the exception of Patient 4. However, given the small sample size of this study, this result was not significant. Further investigation is needed to determine whether the Full VMAT technique in fact results in higher relative dose on average or if this finding is merely coincidental.

**Table 5:** Estimated supraclavicular CTV mean dose gained or lost over the entire course of treatment. Based on hypothetical 60 Gy prescription. Values are in gray

	*Half‐Beam VMAT*	*Full VMAT*
Patient 1	−0.9	0.4
Patient 2	−0.3	1.0
Patient 3	0.1	2.6
Patient 4	1.7	−1.7
Patient 5	−2.0	2.9
Patient 6	−1.0	1.3

The nodal target D95 averaged across all fractions sampled (Fig. 7) was within 2.5% of planned with two exceptions: the Full VMAT plan for Patient 4, and the Half‐Beam VMAT plan for Patient 5. These interesting cases were further investigated.

### A. Patient 4 — Full VMAT

With the Full VMAT plan, Patient 4 showed a marked drop in dose compared to expectation from planning. The nodal target mean dose was on average 2.9% lower than expected, with the worst single fraction being 5.1% lower than expected. The nodal target D95 averaged 6.1% lower than expected, with the worst fraction being 10.4% lower.

The interfractional shoulder motion of Patient 4 was in line with that of the other patients in both average motion and standard deviation. A repeat alignment and contour deformation for the worst single fraction yielded a negligible change in the target mean and D95.

**Figure 8:**
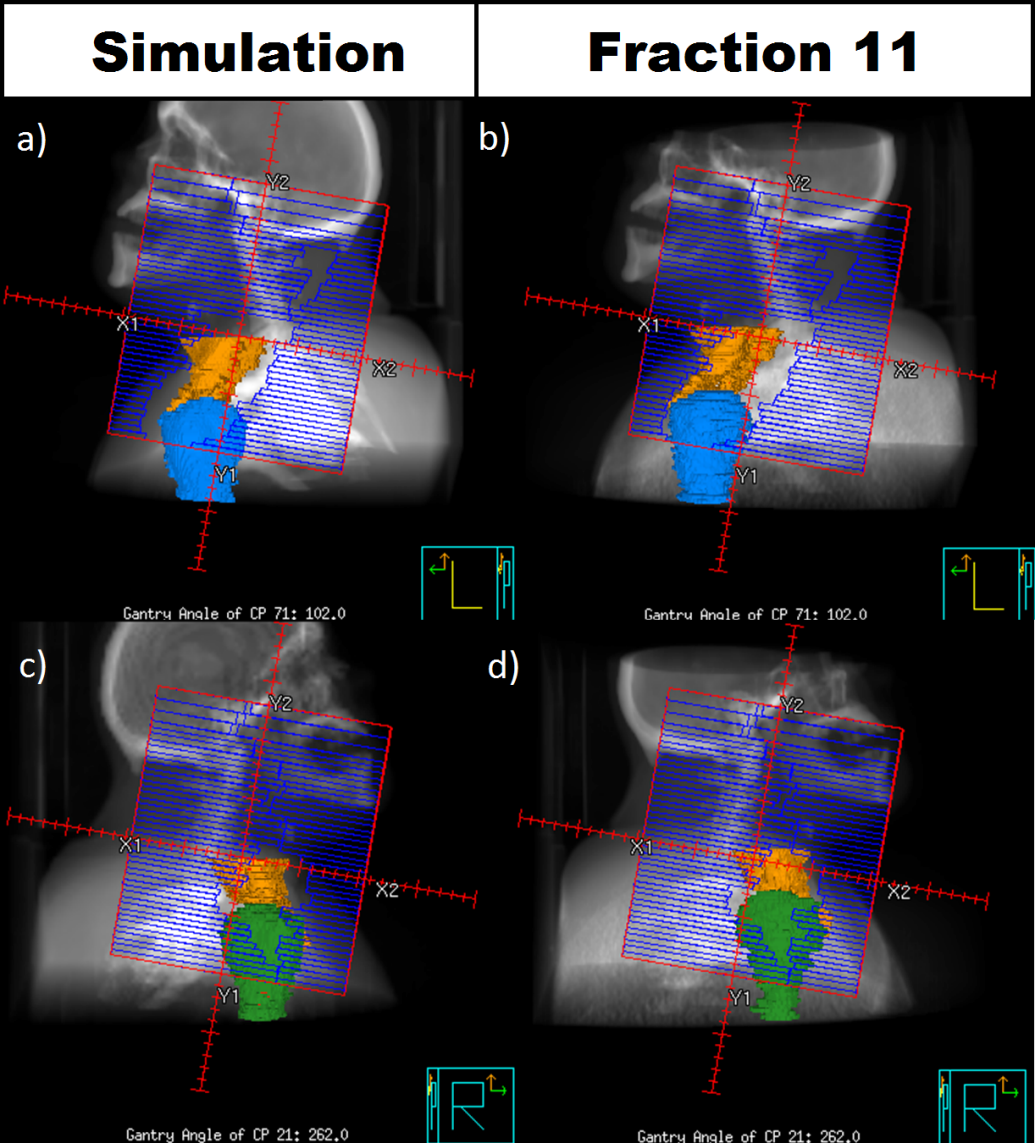
Example left posterior oblique ((a), (b)) and right posterior oblique ((c), (d)) beam's eye views for one arc of the Full VMAT plan for Patient 4. The nodal target is contoured in orange. The left humeral head is contoured in blue and the right humeral head is contoured in green.

The humeral head and proximal humerus of Patient 4 were contoured on both the simulation scan and the weekly CT scan for the worst fraction sampled. Beam's eye views of one VMAT arc at lateral gantry angles are shown in Fig. 8. There was little apparent difference in the amount of the target volume obscured by the humeral head between the simulation CT and the weekly CT. However, an apparent anterior shift in the position of the target volume is visible in the weekly scan (Fig. 8(a) and (b)). We speculate that this movement of the target contributed to the loss of dose observed. It was also notable that Patient 4 had very broad shoulders which were partially truncated in the weekly images by the 50 cm field of view of our CT‐on‐rails. This image truncation may have had an unpredictable effect on the weekly dose calculation for this patient.

### B. Patient 5

Patient 5 also showed a wide disparity between the Full VMAT plan and the Half‐Beam VMAT plan in both mean dose and D95. The average normalized nodal target mean dose was 0.966 for the Half‐Beam VMAT plan and 1.048 for the Full VMAT plan. The average normalized nodal target D95 was 0.955 and 1.013, respectively.

The weekly setup of Patient 5 showed a systematic shift for both shoulders in the posterior direction compared to simulation. However, overriding the density of the humeral heads and proximal humerus to that of water and recalculating the Full VMAT plan resulted in negligible change in the target mean dose.

Interestingly, image registration and contour deformation resulted in a nodal target that was approximately 5 mm deeper to the patient's skin in the weekly images than the simulation images. This effect was likely related to the large shoulder shifts and could have contributed to the dose differences observed, especially for the Half‐Beam VMAT plan, in which the supraclavicular fields were directed only anteroposteriorly.

## V. CONCLUSIONS

We have found interfractional shoulder motion for six patients to be largely consistent with previous literature.^(6)^ Using our immobilization device, most weekly shoulder positions were within 1 cm of the position at the time of simulation in the superior–inferior and anterior–posterior dimensions, with exceptional cases of motion up to approximately 2.5 cm in a single dimension.

We found no clear evidence of superiority in the average supraclavicular target mean dose or average target D95 for either the Full VMAT or the Half‐Beam VMAT technique in six patients. The average target mean dose across all the fractions we sampled was within 5% of that planned at simulation for all patients and all plans. Similarly, the average D95 for the nodal target was also within 5%, with the single exception of the Full VMAT technique for Patient 4, which was 6.1% lower. It was thought that both an anterior shift in the position of the target volume after deformation and the substantial truncation of the patient's shoulders in the weekly CT images contributed to the findings for this particular patient.

On the other hand, the Half‐Beam VMAT technique was clearly superior to the Full VMAT technique, as measured by standard deviation in both target mean dose and D95. The standard deviation in target mean dose was smaller for all six patients with the Half‐Beam VMAT plan; in five of the six cases, the Half‐Beam VMAT target mean dose standard deviation was less than half that of the Full VMAT plan. Similarly for D95, the Half‐Beam VMAT standard deviation was less than that of the Full VMAT plan in four out of six patients. For a fifth patient, the values were comparable (1.8% and 1.7%, respectively).

It is important to note that this analysis assumes a perfect match between the fields in the Half‐Beam plan. This assumption may be unrealistic in light of previous studies^9^, ^10^ and the AAPM Task Group 142 report's^(11)^ suggestion of a 1 mm asymmetric jaw tolerance. Field matching introduces additional uncertainty — which this study did not account for — into treatments in the lower neck region compared to a VMAT‐only approach. This fact should be carefully considered, along with the data presented here, when making decisions regarding treatment techniques.

Finally, the present study made no attempt to measure the effect of different planning techniques on organs at risk (OARs) in the lower neck region. It should not be assumed that OAR doses will be affected in the same way as target dose. However, the topic of OAR dose perturbations is certainly of interest to clinicians when choosing a planning technique, and this presents an intriguing possibility for future study.

In conclusion, it is very difficult to determine a systematic relationship between shoulder position and supraclavicular nodal target dose for any given patient. In fact, any perturbations that may exist due to shoulder position are likely obscured by normal anatomical deformations and movement, reinforcing the need to select appropriate margins during treatment planning. However, we did observe a real difference in the magnitude of interfractional dose variations as measured by standard deviation using two different planning techniques. With this in mind, in the absence of a compelling reason to use a Full VMAT technique and given an understanding and careful consideration of field‐matching uncertainty, the Half‐Beam technique described here likely represents a safer choice for clinicians seeking to minimize interfractional dose variations for patients requiring treatment to nodal targets in the supraclavicular region.

## ACKNOWLEDGMENTS

The authors wish to acknowledge Ryan Williamson at MD Anderson for his assistance with this work.

## Supporting information

Supplementary Material

Supplementary Material

Supplementary Material

Supplementary Material
