# Supplementary figures and images for: Effect of interfractional shoulder motion on low neck nodal targets for patients treated using volumetric‐modulated arc therapy (VMAT)
Source: J Appl Clin Med Phys. 2015 Jul 8;16(4):40–51. doi: 10.1120/jacmp.v16i4.5206 (PMC5690006; doi:10.1120/jacmp.v16i4.5206)

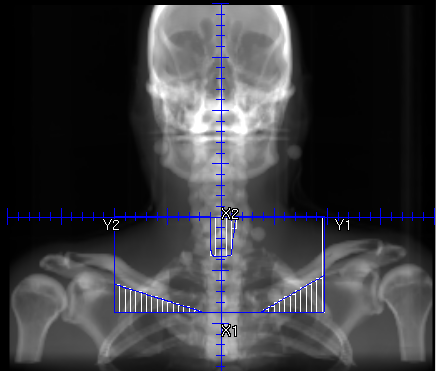

Supplement: Supplementary file 1 — Supplementary Material [file ACM2-16-040-s001.png]

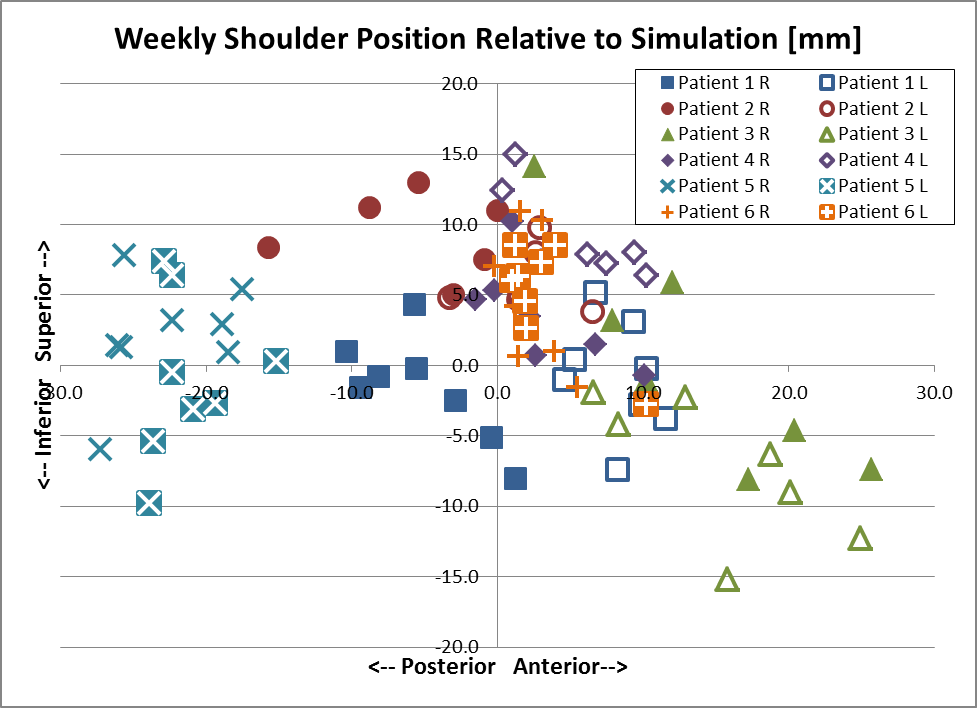

Supplement: Supplementary file 2 — Supplementary Material [file ACM2-16-040-s002.png]

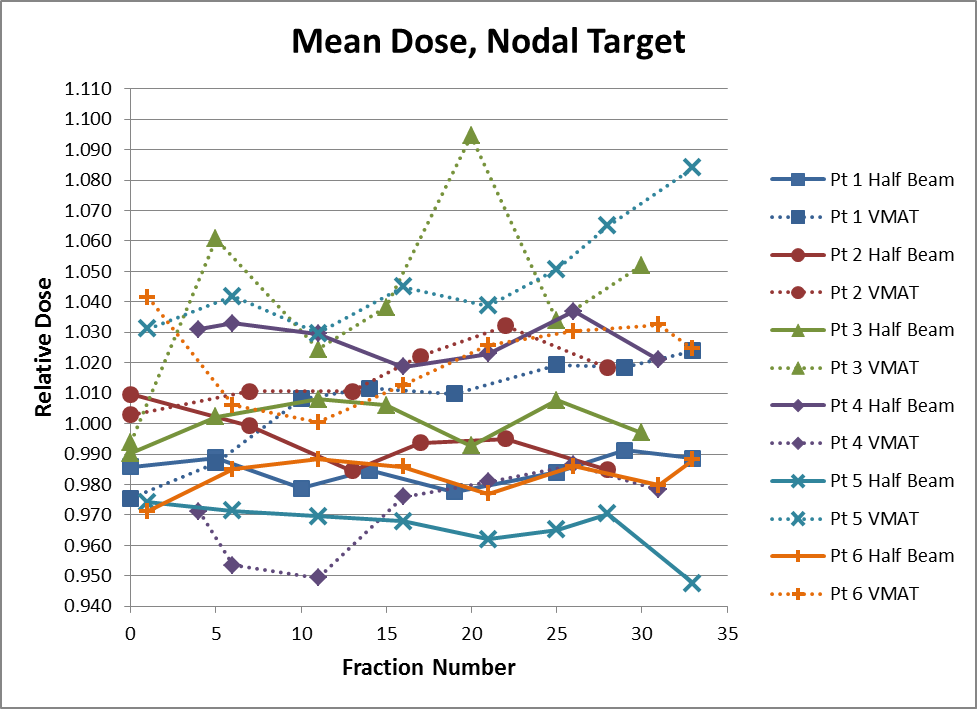

Supplement: Supplementary file 3 — Supplementary Material [file ACM2-16-040-s003.png]

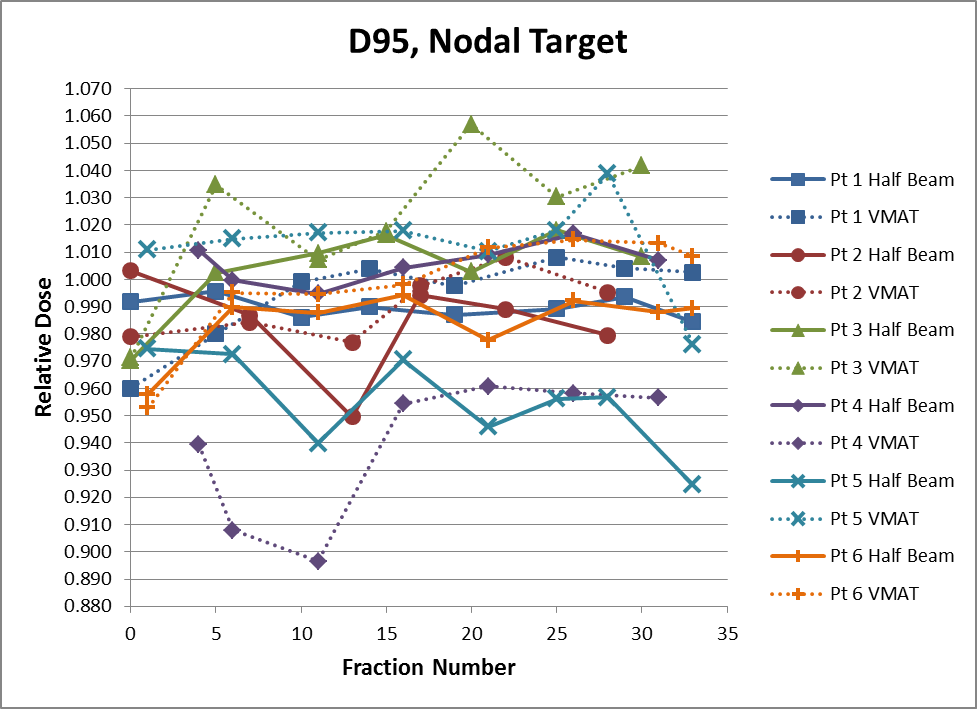

Supplement: Supplementary file 4 — Supplementary Material [file ACM2-16-040-s004.png]
